# Estimating the Analytical Performance of Raman Spectroscopy for Quantification of Active Ingredients in Human *Stratum Corneum*

**DOI:** 10.3390/molecules27092843

**Published:** 2022-04-29

**Authors:** Hichem Kichou, Emilie Munnier, Yuri Dancik, Kamilia Kemel, Hugh J. Byrne, Ali Tfayli, Dominique Bertrand, Martin Soucé, Igor Chourpa, Franck Bonnier

**Affiliations:** 1 Faculté de Pharmacie, Université de Tours, EA6295 NMNS, NanoMédicaments et NanoSondes, 31 Avenue Monge, 37200 Tours, France; hichem.kichou@univ-tours.fr (H.K.); emilie.munnier@univ-tours.fr (E.M.); yuri.dancik@certara.com (Y.D.); kami.kemel@gmail.com (K.K.); martin.czok-souce@univ-tours.fr (M.S.); igor.chourpa@univ-tours.fr (I.C.); 2 Le STUDIUM Institute of Advanced Studies, 1 Rue Dupanloup, 45000 Orléans, France; 3 FOCAS Research Institute, TU Dublin, City Campus, Camden Row, D08 CKP1 Dublin 8, Ireland; hugh.byrne@tudublin.ie; 4 Faculté de Pharmacie, Université Paris-Saclay, Lip(sys)^2^, Lipides, Systèmes Analytiques et Biologiques, 5 Rue Jean-Baptiste Clément, 92290 Châtenay-Malabry, France; ali.tfayli@universite-paris-saclay.fr; 5 Data_Frame, 25 Rue Stendhal, 44300 Nantes, France; dataframe44@gmail.com

**Keywords:** *stratum corneum*, high performance liquid chromatography, confocal Raman spectroscopy, resorcinol, quantification

## Abstract

Confocal Raman microscopy (CRM) has become a versatile technique that can be applied routinely to monitor skin penetration of active molecules. In the present study, CRM coupled to multivariate analysis (namely PLSR—partial least squares regression) is used for the quantitative measurement of an active ingredient (AI) applied to isolated (ex vivo) human *stratum corneum* (SC), using systematically varied doses of resorcinol, as model compound, and the performance is quantified according to key figures of merit defined by regulatory bodies (ICH, FDA, and EMA). A methodology is thus demonstrated to establish the limit of detection (LOD), precision, accuracy, sensitivity (SEN), and selectivity (SEL) of the technique, and the performance according to these key figures of merit is compared to that of similar established methodologies, based on studies available in literature. First, principal components analysis (PCA) was used to examine the variability within the spectral data set collected. Second, ratios calculated from the area under the curve (AUC) of characteristic resorcinol and proteins/lipids bands (1400–1500 cm^−1^) were used to perform linear regression analysis of the Raman spectra. Third, cross-validated PLSR analysis was applied to perform quantitative analysis in the fingerprint region. The AUC results show clearly that the intensities of Raman features in the spectra collected are linearly correlated to resorcinol concentrations in the SC (R^2^ = 0.999) despite a heterogeneity in the distribution of the active molecule in the samples. The Root Mean Square Error of Cross-Validation (RMSECV) (0.017 mg resorcinol/mg SC), The Root Mean Square of Prediction (RMSEP) (0.015 mg resorcinol/mg SC), and R^2^ (0.971) demonstrate the reliability of the linear regression constructed, enabling accurate quantification of resorcinol. Furthermore, the results have enabled the determination, for the first time, of numerical criteria to estimate analytical performances of CRM, including LOD, precision using bias corrected mean square error prediction (BCMSEP), sensitivity, and selectivity, for quantification of the performance of the analytical technique. This is one step further towards demonstrating that Raman spectroscopy complies with international guidelines and to establishing the technique as a reference and approved tool for permeation studies.

## 1. Introduction

One of the most important functions of the skin is as a protective barrier preventing excessive transepidermal water loss while protecting against external physical, chemical, and biological aggressions. The outermost layer of the skin, the *stratum corneum* (SC), is of particular interest, as it determines the effectiveness of this barrier function. A “bricks and mortar” model is commonly used to describe the composition of the SC [1], the corneocyte and corneodesmosome “bricks”, containing highly cross-linked proteins, being held together by an intercellular lipidic matrix consisting of free fatty acids, cholesterol, and ceramides, acting as the ‘mortar’. This specific biochemical architecture of proteins, lipids, and water provides an efficient barrier function [2], which limits the penetration and permeation of exogenous agents [3].

As a direct consequence, active substances (such as active cosmetic ingredient: ACI) and drugs (active pharmaceutical ingredient: API) released from topically applied formulations can also face limited absorption and permeation. It is commonly accepted that the effectiveness of a product is directly correlated to the ability of the ACI or API to penetrate into, and permeate through the skin [4]. Therefore, pharmaceutical and cosmetic industries are driven to develop new formulations, integrating new active molecules into galenic forms with appropriate stability and sensorial properties, but also enabling release and penetration of active ingredients (AI) [5]. Topical formulations, like emulsions or gels, consist of multicomponent mixtures with specific galenic properties. The physicochemical properties of the active ingredients, the rheology of the formulation, the hydrophilic–lipophilic balance and particle size for emulsions are critical parameters which strongly impact on the interaction of the product and the skin, and hence the penetration and permeation [5]. Accordingly, for the development of new formulations, it is necessary to carry out comparative studies to evaluate the efficiency of skin penetration and permeation of an AI [6].

To study skin penetration and permeation ex vivo, data are commonly obtained using Franz diffusion cells coupled to a reference analytical method such as high performance liquid chromatography (HPLC) or UV spectrophotometry [7]. This approach is considered the gold standard, recommended in international guidelines such as the *OECD Guideline 428 and 427 for the testing of chemicals, skin absorption: in vivo and in vitro method* [3], *the Scientific Committee on Consumer Safety* (SCCS) for basic criteria for the in vitro assessment of dermal absorption of cosmetic ingredients [8], and the EMA draft guideline on quality and equivalence of topical products [9]. For this purpose, human biopsies/explants are used to measure the partitioning of an AI into and diffusion within the skin, depending on its physicochemical properties.

In vivo assessment requires more or less invasive protocols to assess the penetration depth of the AI. The so-called tape stripping (TS) approach is widely used to investigate skin penetration of active ingredients into viable tissues [10,11,12]. The method has many limitations, however, including the variability associated with the pressure applied to the tape, the time of contact, and the velocity of removing the tape, leading to uncertainties in the depth of penetration estimated [13].

Microdialysis (MD) is used in dermatological research as an in vivo tool that requires the insertion of a small catheter under the skin to enable measurement of percutaneous drug penetration [10]. This method is a versatile sampling technique that can be used to recover soluble endogenous and exogenous molecules from the extracellular compartment of human skin. MD can be applied in both clinical and preclinical settings [14]. However, the main disadvantage is mechanical stress from the probe that can alter tissue morphology and therefore it can be considered minimally invasive, at best. More recently, open-flow microperfusion (dOFM) has been used as an alternative to MD for its potential for bioequivalence and bioavailability assessment of topical products [15]. However, TS and other methods such as MD and dOFM that are minimally invasive lead to local irritation and discomfort for volunteers.

There is an increasing demand for alternative methods to assess the penetration of active ingredients, ultimately in vivo. Since the pioneering work of Caspers et al. in the 1990s, demonstrating the use of Raman spectroscopy for the molecular analysis of skin structure and composition [16], the technique has been widely documented for its potential in the medical field as a tool for investigation of physio-pathological processes, diagnosis of different skin lesions (atopic skin, cancers, actinic keratosis, etc.) [17,18,19]; in the pharmaceutical and cosmetic fields to investigate the penetration of active molecules [20,21,22,23,24,25]; or to study the effects of UV and pollutants on skin [26]. Confocal Raman microscopy (CRM) is now a versatile technique that can be applied routinely to skin cross sections in vitro, ex vivo on biopsies and in vivo. Operating in confocal conditions, the measurements can be performed from the surface to the deeper layers of skin, non-invasively, to yield information about the water content [16,27], the thickness of skin layers [21,28], study the impact of emulsifiers and formulations on intercellular lipids of *stratum corneum* [29], or to determine the diffusion of active ingredients in the skin [30]. However, Raman spectroscopy has not yet been fully established as a tool for absolute quantification ex vivo or in vivo.

Increasingly, comparative studies of CRM and conventional methods such as TS coupled to HPLC are conducted to highlight the complementarity of the techniques in monitoring AI diffusion into the skin and determining semi-quantitative penetration and permeation profiles [21]. Iliopoulos et al. have carried out a comparative study between in vitro permeation data using Franz cells and in vivo Raman data on niacinamide (NIA). The results demonstrated a good correlation between in vitro cumulative permeation and in vivo skin uptake at −2 µm (R^2^ = 0.98) [31]. Caspers et al. have reported a promising mathematical method for in vivo quantification in the SC, relying on fitting of data collected from skin with Raman spectra collected for reference compounds. This approach was notably applied to calculate the flux of diffusion for trans-retinol after topical applications [32].

Despite the flourish of literature in recent years, few studies have directly targeted the demonstration of analytical performances proscribed by international regulatory authorities such as the US Food and Drug Administration (FDA) [33], the European Medicines Agency (EMA) [34], or the International Council for Harmonisation (ICH) [35], which provide statistical criteria to numerically evaluate a method. Franzen et al. proposed a proof of concept correlating the Raman signal of a model drug with its controlled amount of caffeine in an isolated human SC, determined by CRM and HPLC [36], although without any further insights or discussion about capabilities of the technique for quantification. In a follow-up study, Alonso et al. correlated Raman signal ratios and caffeine concentration obtained by HPLC, but in porcine skin, hence limiting applicability to human skin penetration studies [37].

Therefore, in the present study, CRM coupled with multivariate analysis (namely PLSR—partial least squares regression) is used for the quantitative measurement of resorcinol, selected as a model compound, applied to isolated (ex vivo) human *SC* at systemically varied doses. The performance is quantified according to key figures of merit defined by the regulatory bodies ICH, FDA, and EMA. A methodology is thus demonstrated to establish the limit of detection (LOD), precision, accuracy, sensitivity (SEN), and selectivity (SEL) of the technique, and the performance according to these key figures of merit are compared to similar established methodologies, based on studies available in literature.

## 2. Material and Methods

### 2.1. Chemicals

Resorcinol was purchased from Sigma Aldrich (Saint-Quentin-Fallavier, France), methanol and phosphoric acid from Thermo Fisher Scientific (Iiilkrich-Graffenstaden, France), and phosphate-buffered saline (PBS) from Hyclone Laboratories (Logan, UT, USA). Ultra-pure water was obtained using a Millipore MilliQ system (Merck Millipore, Molsheim, France).

### 2.2. Isolated Human Stratum Corneum

*Stratum corneum* (SC) discs, 12 mm in diameter, were purchased from Biopredic international (Rennes, France). The SC was collected during abdominal skin surgery from a 53-year-old Caucasian female according to the French law L. 1245 CSP “product and element of human body taken during surgical procedure and used for scientific research”. The SC were shipped frozen and were stored at −20 °C until the day of the experiment.

### 2.3. Preparation of Infused SC Samples

On the day of the study, SC were immersed in resorcinol solutions prepared in PBS. Firstly, SC discs were thawed at room temperature for 1 h and weighed. The SC discs were then completely immersed in 4 mL of resorcinol solutions in Petri dishes for 24 h at room temperature, to enable complete infusion until equilibrium.

*Samples for HPLC:* Solutions of 4, 10 and 25 g·L^−1^ resorcinol in PBS were used. For each concentration, 3 SC samples were prepared. After infusion for 24 h, SC were gently removed from resorcinol solutions with tweezers and placed in 2 mL of methanol for 8 h, for extraction of resorcinol, at which point the supernatant was analysed by HPLC.

**Figure 1:**
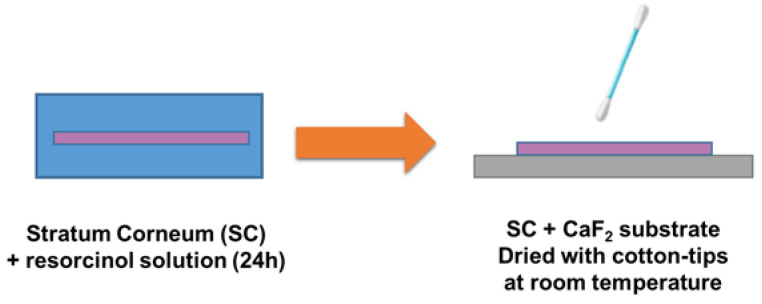
Procedure for infusion of SC samples in resorcinol solutions.

*Samples for Raman spectroscopy:* A range of resorcinol solutions in PBS was prepared for use with SC samples. Concentrations of 0.5, 1, 2.5, 5, 10, 25, and 50 g·L^−1^ are respectively identified as C2, C3, C4, C5, C6, C7, and C8. An SC negative control sample immersed in PBS was additionally prepared (C1). After 24 h immersion, SC discs were collected and deposited on calcium fluoride (CaF_2_) substrates, dried with cotton-tips to remove any excess resorcinol solution on the surface of the SC samples, and then air dried at room temperature before analysis by CRM as described in Figure 1.

### 2.4. HPLC Analysis

HPLC analysis was performed using an Ultimate 3000 (Thermo Fisher Scientific, Voisins-le-Bretonneux, France) piloted by Chromeleon 7.1 software. The system was equipped with a DAD UV ultimate 3000 detector and an C18 column with the particle size of 5 μm and length of 4.6 × 150 mm (Interchim, Montluçon, France). The temperature of the column was adjusted to 25 °C. Absorbance was recorded at 275 nm for detection of resorcinol. The analysis was performed in isocratic mode using a methanol/water (25%/75%) mobile phase supplemented with phosphoric acid at 10 μM. A 10 μL sample of each experimental solution was injected and a flow rate of 1 mL·min^−1^ was applied for 6 min runs per analysis.

*Calibration curve:* To construct the calibration plot, a stock solution of resorcinol was prepared at concentration 1 mg·mL^−1^ in PBS, and serial dilutions were used to obtain the following standard solutions: 0.1, 0.05, 0.01, 0.005, 0.001, 0.0005, and 0.0001 mg·mL^−1^ for analysis by HPLC. The observed retention time was 4.14 min for resorcinol. For each concentration, the area under the peak was calculated and used for the calibration curve [38]. Each day, calibration curves were done in triplicate.

### 2.5. Confocal Raman Microscopy Analysis

CRM analysis was performed using an Alpha300R Raman microscope (WiTec, Ulm, Germany) equipped with 532 nm laser source. To avoid photo damage, the power was set to 10 mW at the samples, corresponding to a laser density of 7.54 mW µm^−2^. A 600 lines/mm grating was selected, and the back scattered light was collected on a back illuminated deep depletion CCD detector over the spectral range 0–3600 cm^−1^ with a spectral resolution ~5 cm^−1^. Spectra were collected using a 20 × objective (Zeiss EC-Epiplan-NEOFLUAR, NA = 0.5, spot size ~1.3 µm) and the acquisition time was set to 30 s × 2 accumulations. For each disc, 20 maps were performed across the SC, to account for spatial heterogeneity. For each map, 9 spectra (3-by-3) were collected using a 4 µm step size, resulting in 180 spectra recorded per sample and a total of 1440 spectra for the study. The instrument is calibrated daily using a 2-step procedure. Firstly, the True Cal function of Project 5 (WITec, Ulm, Germany) is used. It is an automatic multipoint calibration routine performed with a mercury–argon (HgAr) light source integrated in the Raman microscope. Secondly, prior to data acquisition, a verification was done using the peak at 520.7 cm^−1^ from a silicon substrate.

### 2.6. Data Analysis

Data analysis was performed using Matlab^®^ (MathWorks, Natick, MA, USA). The spectra were cut between 400 and 1800 cm^−1^ to refine the analysis to the finger-print region. A Lieber baseline correction was applied [39], using a linear correction function with 10 iterations, followed by a unit vector normalisation.

Principal Components Analysis (PCA): PCA is an unsupervised multivariate analysis technique used to evaluate the variability and to simplify a complex data set of multiple dimensions. It allows the reduction of the number of variables in a multidimensional data set, although it retains most of the variation within the data set. The other advantage of this method is the derivation of PC loadings which represent the variance of each variable (wavenumber) for a given PC, hence reflecting the variations in the chemical components contributing to the spectra [40]. In the scatter plot, the first principal component (PC1) accounts for the highest explained variance. Moreover, each dot in the scatterplot, corresponding to a spectrum, can be located in space using the coordinates defined by the scores along both PC1 and PC2. Therefore, similar spectra tend to be gathered together with relatively close PC scores while the presence of significant variations in spectral features will result in higher distances between spectra. The data collected at a given concentrations are rather grouped in a so-called cluster.

Area under the curve (AUC): The ratio between the AUC 724–794 cm^−1^ corresponding to aromatic ring band of resorcinol and the AUC 1400–1500 cm^−1^ corresponding to C-H of lipids and proteins band was calculated. The value of the band ratio found in the SC control sample C1 (unexposed to resorcinol) was subtracted from all other band ratios calculated from samples C2 to C8.

Partial least squares regression (PLSR): PLSR has been applied to construct a linear regression model. The data set was divided into the training set (3/4 of the Raman maps per sample) and the test set (the remaining 1/4 of Raman maps for each sample). The test set is used as unknown samples to determine the accuracy of the analysis. The training set was further separated in the calibration set (3/4 of samples) and validation sets (1/4 of samples). A 100-fold iteration was implemented to evaluate the stability of the calibration model with multiple random combinations of calibration/validation sets. The validation set is used to select the best number of latent variables, independently of the test set. Notably, in all iterations, the models were tested with the same 1/4 of the data, identified as the test set, i.e., the unknown samples to be determined. The results are presented as predicted concentrations regressed against observed concentrations. The output of this model gives information to evaluate the PLSR using the linearity of the regression between the measured and predicted concentrations (R^2^), root mean square error of cross validation (RMSECV), calculated from the training datasets and the root mean square error of prediction (RMSEP), calculated from the test set. The regression vectors were also used to visualise the variables (wavenumbers) used by the PLSR algorithm that are represented under regression coefficients.

### 2.7. Analytical Performance Figures of Merit

The following figures of merit for performance were used in this study:

*Linearity:* The correlation coefficient (R^2^) is usually used to represent the linearity of a model. For the band ratio of AUC, the R^2^ can be used to evaluate the linearity between the intensity of the instrumental response (peak intensity) as a function of concentration. For PLSR analysis, the R^2^ reflects the linearity between the predicted concentrations regressed against the observed concentration. For both, a value as close as possible to 1 is expected, as a gauge of the reliability of fitting for the linear model. Additionally, for PLSR, the equation of the regression line is expected to display a slope close to 1.

*Precision and accuracy:* The precision is the closeness of agreement among a series of measurements, while the accuracy is the degree of closeness of the measurement between calculated and predicted values. Both were calculated following ICH guides lines [35].

The accuracy from a linear regression can be expressed using the root mean square error (RMSE) (1) [35]:

(1) $$\mathrm{RMSE}=\sqrt{\frac{\sum_{i=1}^{n}{\left(y_{i}-{\;\hat{y}}_{i}\right)}^{2}}{n}}$$

where $y_{i}$ is the measured values (i.e., prepared), ${\;\hat{y}}_{i}$ is the predicted values, and n is the number of samples.

For PLSR, the RMSE is given as the RMSECV or RMSEP calculated respectively for the calibration (training set) and predictive (test set) models. Moreover, the precision of the model can be estimated using bias corrected mean error square prediction (BCMSEP) following Equation (2) [41]. A low BCMSEP corresponds to high model precision.

(2) $$\mathrm{BCMSEP}=\frac{\sum_{i=1}^{n}{\left(y_{i}-{\;\hat{y}}_{i}\right)}^{2}-{\left[\sum_{i=1}^{n}\left(y_{i}-{\;\hat{y}}_{i}\right)\right]}^{2}/n}{n-1}$$

*Limit of detection (LOD):* The IUPAC guidelines define the limit of detection (LOD) as the lowest quantity of analyte that can be distinguished from the blank (absence of analyte) [42]. According to available recommendations from regulatory authorities such as FDA, EMA, and ICH, the LOD from univariate analysis can be calculated as follows:

(3) $${\mathrm{LOD}}_{\mathrm{AUC}}=3.3\;\cdot \;\frac{S_{C1}}{a}$$

where LOD_AUC_ is the lowest detectable concentration calculated, 3.3 is the Student’s t-test value obtained from t-test using a 5% probability and a is the slope of the linear regression model. S_C1_ is defined as the standard deviation of the response and can be determined using two approaches. Firstly, S_C1_ can be the standard deviation of a blank sample, for instance sample C1. Secondly, S_C1_ can be defined as the residual standard deviation of a regression line, i.e., the RMSE. These methods of calculation all estimate deviation in y, i.e., the intensity of the signal measured. In Equation (3), S_C1_ is divided by the slope to convert the value of LOD from Raman intensity to concentrations. The calculation can be extended to PLSR results, by calculating LOD_PLSR_ as follows:

(4) $${\mathrm{LOD}}_{\mathrm{PLSR}}=3.3\;\cdot \;\mathrm{RMSECV}$$

Similar to S_C1_ in Equation (3), RMSE of cross validation (RMSECV) calculated from the training datasets can be determined either including all concentrations C1-C8 analysed or only calculated from the lowest concentration analysed (C1).

*Sensitivity:* According to ICH guidelines, the sensitivity is the lowest analyte concentration that can be measured with acceptable accuracy and precision [35]. The sensitivity was calculated based on the method of Li et al. [43], and the net analyte signal (NAS) developed by Lorber et al. [44], following Equations (5) and (6):

(5) $${\mathrm{SEN}}_{i}=\frac{{\mathrm{NAS}}_{i}}{y_{i}}$$

(6) $${\mathrm{NAS}}_{i}=\left(x_{i}\cdot b\right)\cdot {\left(b^{T}\cdot b\right)}^{-1}\cdot b^{T}$$

The sensitivity (SEN) is reported as a single value averaged for all concentrations analysed, where x_i_ is a sample spectrum, b is a PLSR coefficient column vector, b^T^ is its transposed vector, and y_i_ is the measured concentration of resorcinol in the SC.

*Selectivity:* Selectivity is a measurement of the ability of a method to determine a particular compound in the analysed matrices without interference from other components [35]. The selectivity was calculated based on the method of Li et al. [43], following Equation (7).

(7) $${\mathrm{SEL}}_{i}=\frac{{||\mathrm{NAS}}_{i}||}{\left||x_{i}|\right|}$$

The selectivity (SEL) is reported as an average for all concentrations analysed. With the NAS_i_ vector and x_i_ the sample spectrum.

*Ratio of performance deviation (RPD):* the RPD is the ratio between the standard deviation of measured values from the calibration set, divided by the RMSEP. The higher the RPD value is, the better the performance of model. Values higher than 2.5 are acceptable, values > 5 are adequate for quality control, and values > 10 are considered to be excellent [45]. It is calculated as follows.

(8) $$\mathrm{RPD}=\frac{S_{\mathrm{Ci}}}{\mathrm{RMSEP}}$$

S_Ci_ is the standard deviation for measured values of each concentration from C1 to C8.

*Regression coefficients:* Regression coefficients were also used to visualise the variables (wavenumbers) used by the PLSR algorithm. They can be interpreted similarly to spectra and provide a visual representation of the molecular selectivity.

## 3. Results

### 3.1. Validation of the Protocol for Sample Preparation

**Figure 2:**
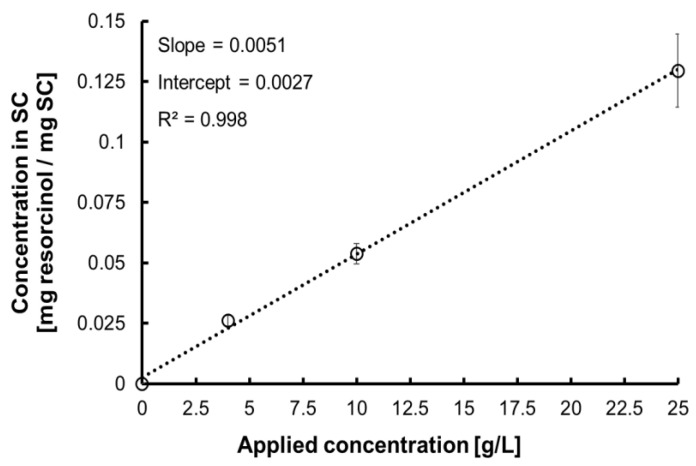
Linear regression of concentration in SC against resorcinol concentrations used as immersion medium.

For the purpose of the study, a protocol that delivers systematically varying mass of resorcinol in SC was developed. Figure 2 shows a plot of the mass (mg) of resorcinol per mass (mg) of SC, as determined by HPLC, regressed against resorcinol concentrations of solutions (g·L^−1^) used as immersion medium. The equation Y=0.0051·X+0.0027 and the R^2^ value 0.998 confirm the proportionality achieved. The analysis validates that SC samples immersed in resorcinol solutions for 24 h reach an equilibrium and that there is a linear relationship between the concentrations of solutions used to immerse SC discs and the resulting concentration in tissues, for instance mg resorcinol/mg SC. Therefore, the linear regression presented in Figure 2 is used as a calibration curve to determine the concentration of resorcinol in SC samples analysed by CRM. Table 1 summarises the concentrations obtained that are used for the subsequent quantitative analysis using univariate and multivariate methods.

### 3.2. Spectral Characterisation of Human Stratum Corneum and Resorcinol by Confocal Raman Microscopy (CRM)

**Figure 3:**
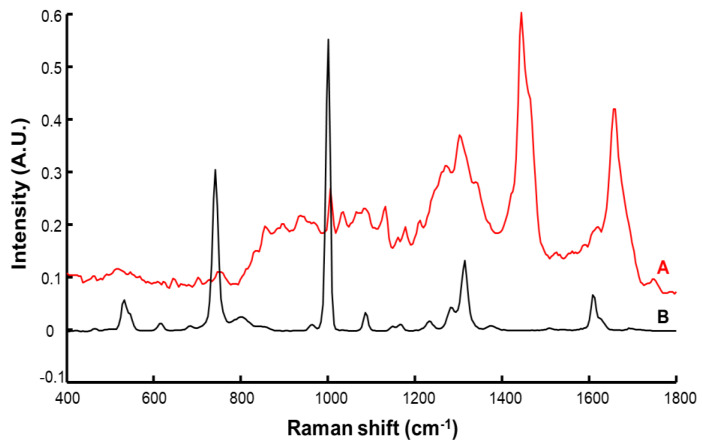
Raman spectra of control *stratum corneum* (A) and pure resorcinol (B). Spectra are offset for clarity.

Figure 3A displays the mean spectrum of control SC samples (i.e., C1). The main characteristic features observed originate from the lipids, nucleic acids, and proteins found in human skin [27]. The broad band at 1655 cm^−1^ is assigned to amide I of proteins (N–C=O deformation). The intense band between 1440 and 1469 cm^−1^ is assigned to combined contributions of C–H scissoring from proteins and lipids. However, the position of the maximum at 1444 cm^−1^ and the presence of a shoulder at 1465 cm^−1^ suggests a high lipid content [46,47]. This is confirmed by the bands found at 1303 cm^−1^ and 1750 cm^−1^, respectively assigned to CH_2_ deformation and C–O–O elongation in lipids [46]. The sharp peak at 1005 cm^−1^ is typically found in biological samples and assigned to the ring breathing (C–C=C deformation) of aromatic acids (phenylalanine) [47,48]. Table 2 provides a list of bands reported in the literature for the SC [46,47,48,49].

The Raman spectrum collected from resorcinol (Figure 3B) displays fewer sharp bands. Notably, the strongest features found are assigned to aromatic ring deformation at 532 cm^−1^, C–C stretching of the aromatic ring at 741 cm^−1^ and 1001 cm^−1^, O–H deformation at 1314 cm^−1^, and C–C stretching at 1608 cm^−1^. Further weaker features are assigned to aromatic ring twisting at 617 cm^−1^, C–C of aromatic ring elongation at 1086 cm^−1^, and C–H in plane bending of the aromatic ring at 1186 cm^−1^ [50].

**Figure 4:**
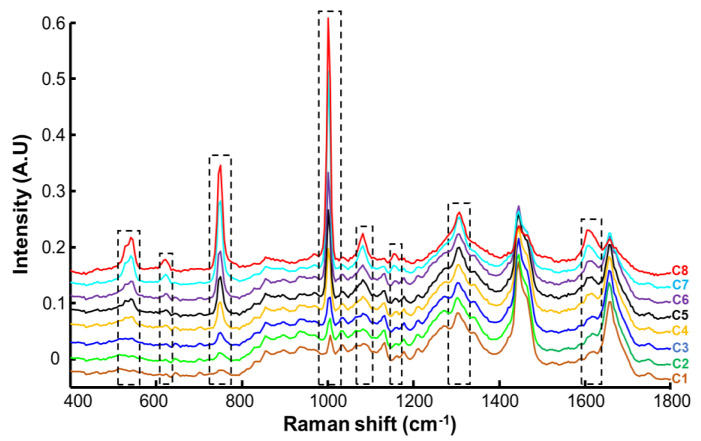
Raman spectra of control SC (C1) and SC infused with resorcinol. a: C2—0.006 mg resorcinol/mg SC; b: C3—0.008 mg resorcinol/mg SC; c: C4—0.016 mg resorcinol/mg SC; d: C5—0.028 mg resorcinol/mg SC; e: C6—0.054 mg resorcinol/mg SC; f: C7—0.130 mg resorcinol/mg SC; and g: C8—0.257 mg resorcinol/mg SC. Spectra are offset for clarity.

Figure 4 shows the mean Raman spectra corresponding to control SC (C1) and SC infused with resorcinol solutions at different concentration (C2 to C8) for 8 h. Raman spectra from immersed SC show that specific bands of resorcinol increase in the *SC* according to the concentration of resorcinol solutions, most evident for the bands at 532 cm^−1^, 610 cm^−1^, and 741 cm^−1^, which are in a spectral region in which strong features from skin constituents are absent. Vibrational modes like the ring breathing (C–C=C deformation) of aromatic acids can be shared between SC and resorcinol, although the peak positions from sample C1 to C8 remain specific to the chemical composition. Notably, the peak observed at 1005 cm^−1^ in the control sample (C1) gradually shifts to 1001 cm^−1^ as the concentration of resorcinol increases (C2–C8), also coupled to a significant increase of the band intensity. This is also observed for other features from resorcinol at 1086 cm^−1^, 1186 cm^−1^, 1314 cm^−1^, and 1608 cm^−1^ that display variations in positions and intensities that can also be correlated with the variations in resorcinol concentration in SC. Although they overlap with bands arising from the strong contributions of endogenous proteins and lipids in this spectral range of the fingerprint region, when the concentration of the resorcinol in the tissue increases, it gradually dominates the spectral signatures collected (C8).

### 3.3. Data Exploration by Principal Component Analysis (PCA)

**Figure 5:**
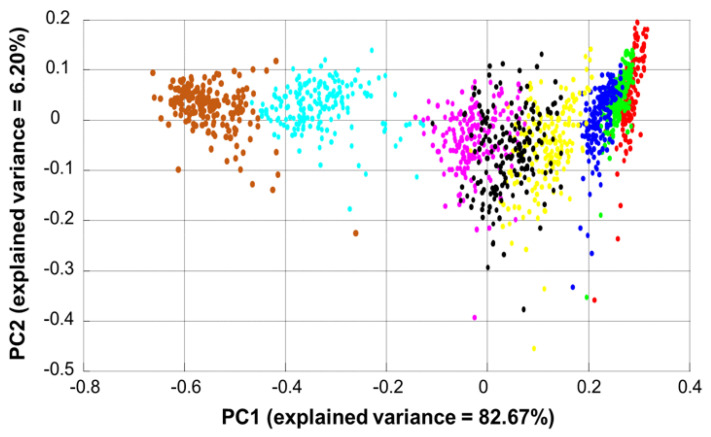
Scatter plot for the first two components obtained from PCA. Red is Raman spectra of control SC (C1), green is C2—0.006 mg resorcinol/mg SC, blue C3—0.008 mg resorcinol/mg SC, yellow C4—0.016 mg resorcinol/mg SC, black is C5—0.028 mg resorcinol/mg SC, magenta is C6—0.054 in turquoise is C7—0.130 mg resorcinol/mg SC, and brown is C8—0.257 mg resorcinol/mg SC.

**Figure 6:**
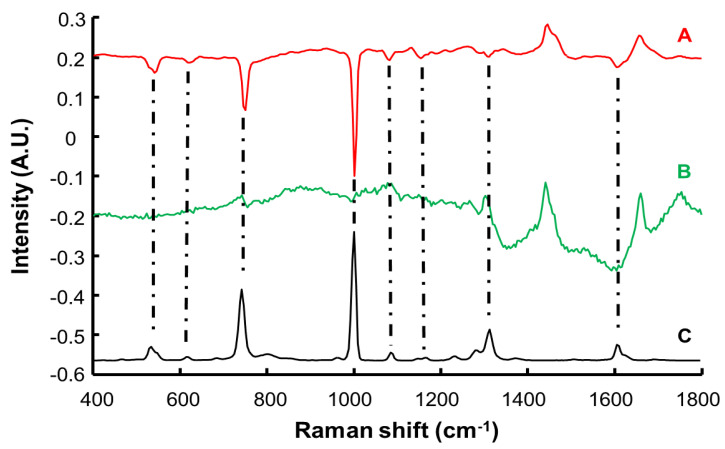
Loadings plot for the two first components obtained from PCA and resorcinol spectrum. A: loading of PC1, B: loading of PC2 and C: reference spectrum from resorcinol (powder).

The scatter plot in Figure 5 shows the distribution of data along PC1 (82.67% of the explained variance) and PC2 (6.20% of the explained variance). Along PC1, data are organised according to the concentration of resorcinol in SC samples starting from left with C8 (brown dot, 0.257 mg resorcinol/mg SC) to the right with C0 (red dots, control C0). Loading 1, shown in Figure 6A, highlights that PC1 reflects spectral variations specific to resorcinol. It is clearly observed that negative peaks of the loading 1 at 532 cm^−1^, 617 cm^−1^, 741 cm^−1^, 1001 cm^−1^, 1086 cm^−1^, 1186 cm^−1^, 1314 cm^−1^, and 1608 cm^−1^ match with peaks identified from the pure compound (Figure 6A,C—dotted lines). Interestingly, weaker features of resorcinol, overlapping with SC constituents at 617 cm^−1^, 1086 cm^−1^, 1186 cm^−1^, and 1314 cm^−1^ (Figure 3), are observed in the loading of PC1, indicating that they also contribute to the variance of the data along PC1. The positive features are assigned to biochemical constituents of the SC (Figure 6B) which are listed in Table 2.

PCA is a powerful tool to illustrate the spectral variability and detect subtle modifications in features. While PC1 captures the systematic variability of the samples infused with different AI concentrations, it also indicates a degree of intra-sample variability. Skin is a complex biological matrix, and it can be anticipated that the distribution of resorcinol across the *SC* would display a level of heterogeneity, inherent in biological systems which are heterogeneous by nature. The heterogeneous structure of the corneocytes embedded in the lipid matrix of the SC results in an uneven distribution of the hydrophilic resorcinol (log K_OW_ = 0.78, octanol/water partition coefficient [51]). Although the data have been recorded with a 20 × objective to increase the spot size to ~1.3 µm, it is natural to observe such spectral variation. Indeed, the purpose of the study is the calculation of the analytical performances specifically for quantification in the SC. Therefore, the variability linked to biochemical properties is encompassed within the data collected.

The loading of PC2, shown in Figure 6B, reflects the further variability of biological heterogeneity specific to SC. The most intense and positive peaks, observed at 1303 cm^−1^, 1444 cm^−1^, 1655 cm^−1^, and 1750 cm^−1^, correspond to features of SC (Figure 3A). It is seen in Figure 5 that the distribution along PC2 is consistent for all concentrations and that all the clusters are aligned, with no possible discrimination along this principal component. Therefore, the variability along the second principal component, PC2, is not related to resorcinol (Figure 6B), but represents intra-sample biological variability.

### 3.4. Data Analysis Using Area under the Curve

**Figure 7:**
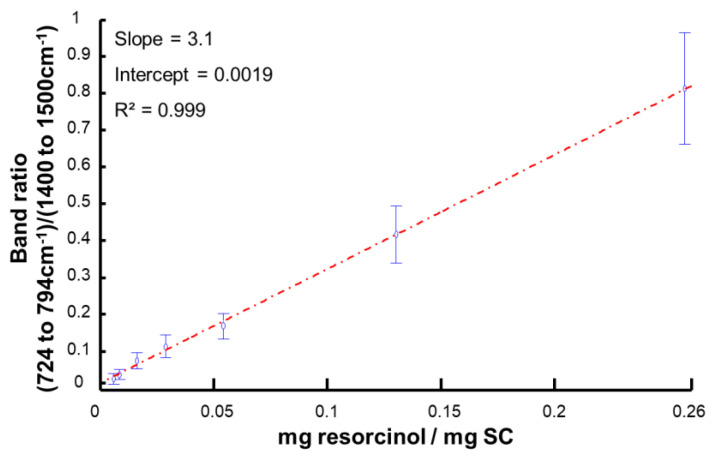
Band ratio of the AUC of the C–C band of the aromatic resorcinol ring [724–794 cm^−1^] and the C–H band of protein/lipids of the SC [1400–1500 cm^−1^] regressed against resorcinol content in SC expressed as mg resorcinol/mg SC.

Figure 7 shows the linear regression constructed using the integrated band ratio of AUC of the feature of SC (1400–1500 cm^−1^) to that of resorcinol (724–794 cm^−1^). The regression has a linearity characterised by a value of R^2^ = 0.999. The proportionality between the band ratio calculated from Raman spectra and the concentration of resorcinol in SC samples confirms the suitability of the immersion setup as an in vitro model that can be used to simulate different levels of diffusion within the skin tissues. The variations in intensities for features of resorcinol in Raman spectra linearly correlates with the content expressed as mg resorcinol/mg SC. The error bars, representing the standard deviation for each concentration, clearly highlight the spectral variability in the data sets, as observed previously with PCA (Figure 5). The RMSE calculated from Figure 7 was found to be 0.011 mg resorcinol/mg SC. According to Equation (1), the difference between $y_{i}$, the measured values (i.e., prepared), and ${\;\hat{y}}_{i}$, the predicted values, is taken into account, and therefore the results represent the performances of Raman spectroscopy in the SC, taking into account the biological and biochemical heterogeneities.

The limit of detection (LOD) is one of the most important key figures of merit to be determined to evaluate analytical methods, as it depends on both the precision and sensitivity of the method [52,53]. According to the definition of the International Standardization Organization (ISO), adopted by the International Union of Pure Applied Chemistry (IUPAC), the LOD indicates the lowest quantity of an analyte which can be distinguished from the absence of the analyte within a stated confidence limit [54]. According to Equation (3), the LOD_AUC_ can differ depending on the determination of the parameter S_C1_.

When S_C1_ is determined as the standard deviation from blank samples, for instance sample C1, S_C1_ = 0.016 mg resorcinol/mg SC and the LOD_AUC_ = 0.017 mg resorcinol/mg SC. Alternatively, when S_C1_ is determined as the residual standard deviation (i.e., RMSE) of the regression line from Figure 7, S_C1_ = 0.011 mg resorcinol/mg SC and LOD_AUC_ = 0.012 mg resorcinol/mg SC. Although similar, the LOD_AUC_ value calculated from the blank sample is 1.4 times higher than the LOD_AUC_ value using the RMSE. While AUC is a rapid approach to visualise variations in data sets, the calculation can be influenced by residual contribution from the SC, notably for the resorcinol band (724–794 cm^−1^). Multivariate methods of determining the LOD, using the full range (for instance the fingerprint region) may be more appropriate to delivery more specific and accurate figures of merits describing the analytical performances of Raman spectroscopy. To this end, partial least squares regression (PLSR) was applied to the data set.

### 3.5. Data Analysis Using Partial Least Squares Regression (PLSR)

**Figure 8:**
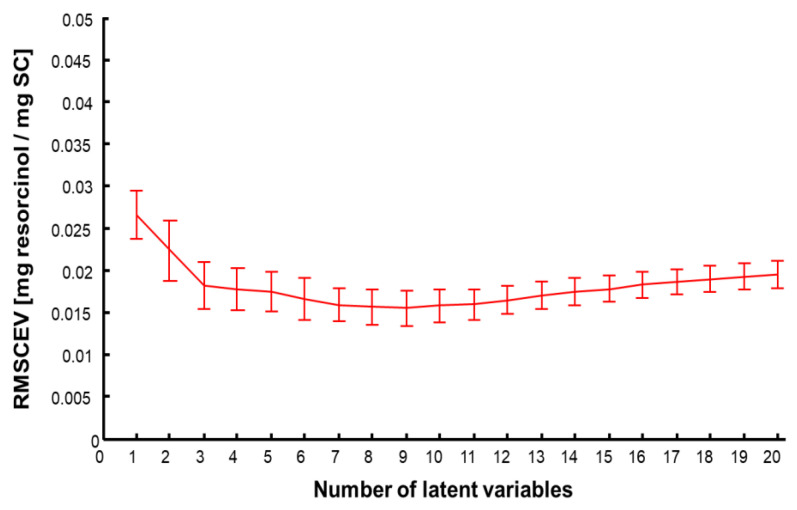
Root mean square error of cross validation (RMSECV).

**Figure 9:**
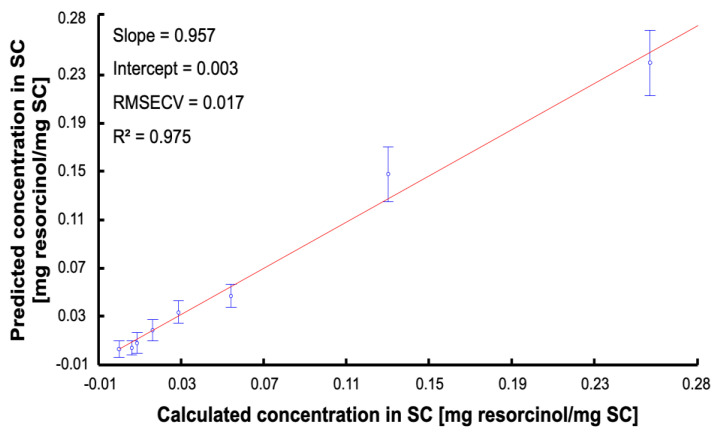
PLSR plot for the validation set. Dots are mean predicted concentration and errors bar represent the standard deviation.

**Figure 10:**
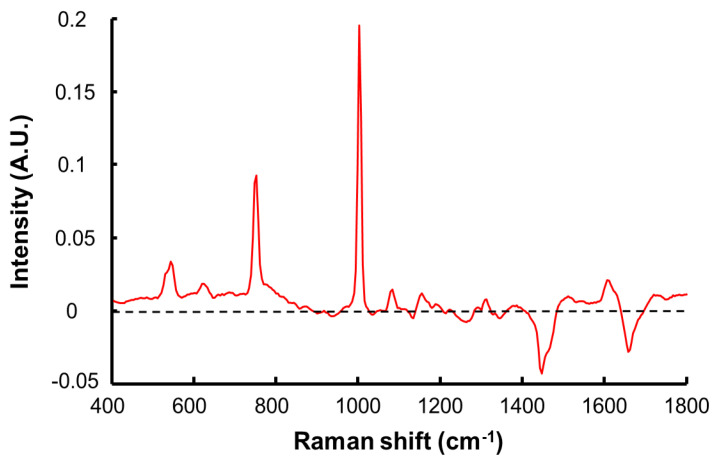
First regression coefficient from the PLSR analysis.

PLSR is the most widely used multivariate method to perform quantitative analysis of spectral data sets [43,55,56]. The first step is to construct the predictive model using a training set corresponding to 3/4 of the maps analysed for each SC sample. A cross validation was applied using 2/3 of the data from the training set used as the calibration set and the remaining 1/3 of the dataset as the validation set. Figure 8 displays the RMSECV as a function of number of latent variables (LV). There is a slight decrease in the RMSECV for the three first LV, whereafter the overlapping errors bars suggests the improvement in the accuracy of the model is not significant. Therefore, it was found preferable to select n = 3 for the LV to avoid introducing noise or interferences in the calibration model. The PLSR plot for the cross validation, i.e., predicted concentrations in SC samples regressed against the calculated (reference) concentrations, is presented in Figure 9. For each concentration, C1–C8, results are provided as mean predicted concentration and errors bars represent standard deviations. The linear regression gives an equation Y = 0.957·X + 0.003 and R^2^ = 0.975. The slope close to 1 suggests a good correlation between prepared and predicted concentrations is observed. The R^2^ inferior to 0.99 is due to the large error bars that are linked to the intra-sample heterogeneity. The value of RMSECV = 0.017 mg resorcinol/mg SC corresponds to 13.2% of the median concentration of the range studied. The regression coefficient (Figure 10) highlights the spectral characteristics used for the construction of the quantitative model using PLSR. It is clearly observed that intense positive features at 532 cm^−1^, 617 cm^−1^, 741 cm^−1^, 1001 cm^−1^, 1086 cm^−1^, 1186 cm^−1^, 1314 cm^−1^, and 1608 cm^−1^ seen in Figure 10 match well with resorcinol bands (see Figure 3B). Negative features at 1655 cm^−1^ and 1444 cm^−1^ are characteristic of the biochemical constituents found in the SC (Table 2).

**Figure 11:**
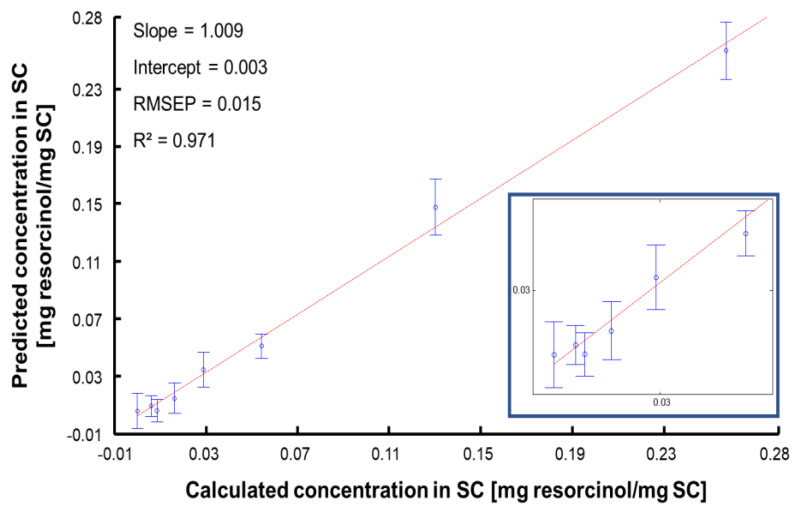
PLSR plot for the test set. Dots are mean predicted concentration and errors bars represent the standard deviation.

The second step of PLSR is to evaluate the quantitative performances of the PLSR model with a test set. For this purpose, 1/4 of the Raman maps analysed for each SC sample was kept independent and projected in the predictive models as unknown samples to be determined. As described in Section 2.3, the samples in the test set are also prepared by immersion and hence their concentration is known; these values are not used during the training of PLSR model in order not to bias the analysis, but rather at the later stage to assess the performance of the model based on the RMSEP and R^2^. The PLSR plot obtained from the test set is presented in Figure 11. The linear regression gives an equation Y = 1.009·X + 0.003 and a R^2^ = 0.971. The RMSEP = 0.015 mg resorcinol/mg SC corresponds to 11.67% compared to the median concentration of the range studied.

While RMECV, RMSEP, and the respective R^2^ are commonly used to interpret PLSR outcomes, additional figures of merit referred to in guidelines and the literature are available to evaluate statistically the performance of an analytical technique. Multivariate data analysis generates more complex modes to interpret especially for data collected from biological systems like SC, because the data mining is performed using all wavenumbers from a given spectral range (for instance fingerprint region), hence including more variables in the calculations [52,57,58].

To enable comparison with results obtained from AUC in the previous section, LOD_PLSR_ was determined using the RMSECV from the blank sample C1 (LOD_PLSR_ = 0.023 mg resorcinol/mg SC) and using the RMSECV including concentrations C1–C8 (LOD_PLSR_ = 0.06 mg resorcinol/mg SC). LOD_PLSR_ using the RMSECV, including all concentrations, is 2.6 times higher compared to the LOD_PLSR_ using the RMSECV from the blank sample C1. This difference can be simply explained by the origin of the errors accounted for. For the blank sample, C1, the variability in Raman spectra collected is solely attributed to biological heterogeneity, i.e., variations mostly due to proteins/lipids and other skin constituents contributing to the data collected. However, for C2–C8, in addition to the biological heterogeneity, there is the heterogeneity of resorcinol distribution across the SC (see PCA scatter plot in Figure 5). This observation is confirmed in Figure 7 by the increasing standard deviation from rationed AUC as a function of concentration. Therefore, the LOD_PLSR_ = 0.06 mg resorcinol/mg SC, incorporating all sources of errors in the calculation, better reflects the performance of Raman spectroscopy applied to the quantification of an active molecule in a complex biological system. Furthermore, the LOD_AUC_ = 0.012 to 0.017 mg resorcinol/mg SC is ≈3.5 times lower than LOD_PLSR_. The difference can be explained by PLSR being more challenging to the data due to the three-way splitting into calibration, validation, and test sets.

PLSR results can also be assessed according to various statistical criteria, described in Section 2, including ratio of performance deviation (RPD), precision (BCMSEP), sensitivity (SEN), and selectivity (SEL) (Table 3). The value of RPD = 8.112, that is superior to 5, indicates a good model performance, while the BCMSEP value of 5.15 × 10^−5^ mg resorcinol/mg SC, representing 0.04% of the range of concentration studied, highlights the potential of Raman spectroscopy to accurately determine the amount of resorcinol in the skin, over the range extending from 0.006 mg resorcinol/mg SC to 0.257 mg resorcinol/mg SC. The sensitivity (SEN) is a representation of the lowest analyte concentration that can be measured with acceptable accuracy and precision [35]. The higher the SEN value is, the better the detection of compounds and therefore the performance of the technique. The value of SEN = 18.994 mg SC · mg of resorcinol^−1^ suggests a quite high sensitivity. However, SEL = 0.3 clearly indicates a lower selectivity. According to the ICH, the selectivity (SEL) is the ability of an analytical method to differentiate and measure the analyte in the presence of potential interfering substances in the blank biological matrix [35]. SEL is a dimensionless criterion ranging between 0 and 1. A value of SEL = 0 implies complete overlap between analyte and interferences. SEL = 1 indicates no overlap and that the model is more selective [56]. The relatively low value obtained is consistent with observation made in Figure 4 and Figure 5 of the degree of interference and intrinsic variability of the spectral features of the SC matrix. While mean spectra indicate that features from SC are broad peaks overlapping with specific bands from resorcinol, PCA highlighted the presence of an important biological heterogeneity that also impact on the selectivity. SC is a complex matrix with proteins, nucleic acids, and lipids from different classes (ceramides, phospholipids, etc.) resulting in a strong Raman response with numerous broad bands that overlap with the features from the analyte (resorcinol), like the aromatic ring deformation at 532 cm^−1^, C–C stretching of the aromatic ring at 741 cm^−1^ and 1001 cm^−1^ which interfere/overlap with C–C deformation and stretching and of phenylalanine ring at 614 cm^−1^, 751 cm^−1^, and 1005 cm^−1^. Furthermore, in Figure 10, the regression coefficient from PLSR enables identification of specific features from resorcinol (positive peaks), but also demonstrates that the quantification relies also on the SC features (negative peaks).

## 4. Discussion

The study of human skin represents an important area of research and development in many areas, including dermatology, toxicology, pharmacology, and cosmetology. In this context, a method which can noninvasively and quantitatively determine the concentration profile of an AI in the skin, in situ, is highly desirable. A number of studies have clearly demonstrated that confocal Raman microscopy can effectively profile the chemical composition of skin, and monitor the penetration and permeation of topically-applied exogenous molecules/agents both in vitro and in vivo [21,30]. Critical to its legislative acceptance as a methodology for routine screening of AI penetration and permeation in skin is the standardisation of protocols and the quantification and optimisation of performance according to key figures of merit defined by legislative/regulatory bodies.

However, there are few reported studies about quantification of active molecules in human skin using Raman spectroscopy that provide reference values for these criteria. Although a few studies, for example Caspers et al. [32] and Iliopoulos et al. [31], have demonstrated the ability of CRM to quantify the permeated flux [22], criteria such as RPD, BCMSEP, SEN, and SEL are essential not only to evaluate the performance of a technique but also to enable direct comparison of studies from the literature. Unfortunately, these key figures of merit are seldom considered in studies reporting quantitative application of Raman spectroscopy, not only for skin analysis, but in general.

Applying Raman spectroscopy to quantify ingredients in pharmaceutical tablets, Short et al. reported values of SEN = 15.13 (%^−1^) and SEL = 0.37 for theophylline, SEN = 17.31 (%^−1^) and SEL = 0.37 for lactose, SEN = 11.85 (%^−1^) and SEL = 0.18 for microcrystalline cellulose, and SEN = 10.53 (%^−1^) and SEL = 0.16 for starch, although no values of RPD and BCMSEP were reported [59]. The consistently high values of SEN, but low SEL, are similar to the observations for AI in SC reported here, as the solid matrix of the tablets analysed contributes a high degree of variability. Schönbichler et al. quantified furosemide from a powder mixture using Raman spectroscopy with a RPD value between 6.67 and 9.13, depending on data pre-processing [45]. Joshi et al. obtained a RPD = 7.84 and SEL = 0.01 for the quantification of olive oil in argan oil matrix using Raman spectroscopy [60]. In both these studies, it is also observed that RPD reported for Raman are within the range 5 and 10, considered adequate for quality control. Fan et al. have used surface-enhanced Raman spectroscopy (SERS) for quantification of carbaryl pesticides in Fuji apples with a RPD equal to 7.71 [61]. Despite the enhancement of the Raman signals, the RPD value did not improve compared to other studies.

Li et al. have reported in situ Raman spectroscopy for real time quantification of cell culture medium in a bioreactor to monitor the production of a monoclonal antibody [43]. RPD values for key parameters were 4.8 for the antibody, 8.1 for glucose, 2.5 for lactate, and 2.6 for glutamine. It was observed that the RPDs display significant variations, depending on the compounds quantified, which can have higher or lower Raman scattering cross sections (Raman intensity). The dynamic range of concentrations found in the mixtures was also seen to be a contributing factor. For instance, glucose, with the highest RPD, also had the broadest range of concentration 0–4.5 g·L^−1^. BCMSEP further reflects the importance of concentration ranges to interpret the results. Li et al. reported values equal to 4.43 × 10^−4^ g·L^−1^ for the antibody, 0.189 g·L^−1^ for the glucose, 0.4716 g·L^−1^ for lactate, and 0.026 g·L^−1^ for glutamine [43]. However, when these values are expressed as percentage of the concentration range analysed, it is found that BCMSEP represent respectively 0.22%, 8.4%, 41.9%, and 89.65% for the antibody, glucose, lactate, and glutamine. It suggests that, despite the low absolute value of BCMSEP obtained for glutamine, the precision is quite poor when concentrations analysed are considered. SEN values were respectively 14.24 g·L^−1^ for the antibody, 0.28 mM^−1^ for glucose, 0.67 mM^−1^ for lactate, and 1.43 mM^−1^ for glutamine. In the study of Li et al., SEL values of 0.65 for the antibody, 0.72 for glucose, 0.74 for lactate, and 0.67 for glutamine were determined. All values above 0.5 are not surprising, due to the difference in Raman spectral signatures collected from these compounds.

Therefore, although reports are few, it can be deduced that the reliability of the predictive model is significantly influenced by the complexity of the matrix analysed, the range of concentration studied, the strength of the Raman response of the AI, all of which can contribute to the degree of heterogeneity in the data collected. Coupling Raman spectroscopy with multivariate data mining methods such as PLSR enables the variance to be captured, reduced, and quantified.

## 5. Conclusions

Raman spectra encompass biochemical information about skin composition and the presence of active ingredients. Performing quantitative analysis for monitoring their penetration and diffusion in the skin relies on the sensitivity and specificity of the technique. In order to determine key figures of merit to characterise the performance of confocal Raman microscopy (CRM), an in vitro model delivering controlled concentrations in human *stratum corneum* (SC) has been developed. Resorcinol, used as model compound, can be detected and quantified in isolated SC samples coupling CRM to multivariate PLSR analysis. The RMSECV (0.017 mg resorcinol/mg SC), the RMSEP (0.015 mg resorcinol/mg SC), and R^2^ (0.971) demonstrate the reliability of the linear regression constructed enable accurate quantification of resorcinol. Furthermore, the results have enabled the determination, for the first time, of numerical criteria to estimate analytical performances of CRM, including limit of detection (LOD), precision (BCMSEP), sensitivity (SEN), and selectivity (SEL) for quantification of the performance of the analytical technique. This is one crucial step towards demonstrating the compliance of Raman spectroscopy with international guidelines and towards establishing the technique as a reference and approved tool for permeation studies.

## Figures and Tables

**Table 1:** Calculated reference concentrations in SC for samples analysed by CRM.

	Applied Concentration [g·L^−1^]	Concentration in SC [mg resorcinol/mg SC]
C1	0	0
C2	0.5	0.006
C3	1	0.008
C4	2.5	0.016
C5	5	0.028
C6	10	0.054
C7	25	0.130
C8	50	0.257

**Table 2:** Raman band assignments in SC [46,47,48,49].

Raman Shift (cm^−1^)	Assignment
**614**	Phenylalanine ring
**646**	Tyrosine ring, ν C–S
**702**	ν C–S gauche of cysteine cholesterol
**751**	Aromatic ring puckering
**864**	Proline, RNA
**898**	Tryptophan
**939**	ρ CH_3_ terminal, m CC a helix (secondary structure), phospholipids
**1005**	Symmetric ring breathing phenylalanine
**1033**	C–H Phenylalanine
**1067**	Lipids: skeletal trans conformation ceramides; ν
**1130**	Lipids: hydrocarbon chain, trans conformation ceramides
**1177**	CH Tyrosine, phenylalanine
**1303**	Amide III, CH_2_ phospholipids
**1444**	δ CH proteins and lipids
**1524**	–C=C– carotenoids
**1655**	Amide I

ν: stretch; δ: deformation; ρ: rocking.

**Table 3:** Estimation of analytical performance parameters of the models using PLSR data (RMSECV, RMSEP, and BCMSEP are in mg resorcinol/mg SC, sensitivity is in (mg SC·mg of resorcinol^−1^), and all other parameters are dimensionless).

***RMSECV***	0.017
***RMSEP***	0.015
***Linearity (R^2^)***	0.971
***RPD***	8.112
***BCMSEP***	5.15 × 10^−5^
***Sensitivity (sen)***	18.994
***Selectivity (sel)***	0.3

## Data Availability

The data presented in this study are available on request from the corresponding author.

## Sample Availability

Samples of the compounds are not available from the authors.
